# The complications of traditional uvulectomy and concurrent occurrences of cultural malpractices in Ethiopia: A systematic review and meta-analysis

**DOI:** 10.1016/j.heliyon.2024.e38978

**Published:** 2024-10-05

**Authors:** Tamirat Getachew, Abraham Negash, Sinetibeb Mesfin Kebede, Abera Cheru, Addis Eyeberu, Abera Kenay Tura

**Affiliations:** a School of Nursing and Midwifery, College of Health and Medical Sciences, Haramaya University, Harar, Ethiopia; b School of Environmental Health Sciences, College of Health and Medical Sciences, Haramaya University, Harar, Ethiopia

**Keywords:** Uvulectomy, Traditional/cultural malpractices, Concurrent occurrence, Ethiopia

## Abstract

**Introduction:**

Despite the establishment of a national strategy and plan to eliminate all harmful traditional practices, traditional uvulectomy remains widely practiced in Ethiopia, and there is a lack of comprehensive summary of national data on uvulectomy complications and associated malpractices. Therefore, this study aimed to assess the pooled complications of uvulectomy and concurrent occurrences of traditional malpractices in Ethiopia.

**Methods:**

The following databases were used to retrieve studies: PubMed, EMBASE, CINAHL, Google Scholar, Web of Science, MEDLINE, Cochrane Library, SCOPUS, and Google Search. Manual searches were also utilized to identify relevant articles. Studies reporting traditional uvulectomy complications and malpractices in Ethiopia were considered. STATA Version 17 was utilized for statistical analyses, while heterogeneity and publication bias were assessed using I^2^ statistics.

**Results:**

From a total of 259 studies found in electronic databases, 19 studies (23,559 study participants) were incorporated in the analysis. The pooled incidence of complications following traditional uvulectomy in Ethiopia was 29 % (95 % CI: 14%–44 %). Hemorrhage, transmission of communicable infections, and sepsis are the most common complications of traditional uvulectomy. Concurrent to uvulectomy female genital cutting (23 %), milk tooth extraction (29 %), bloodletting (“mebtat” in Amharic) (11 %), eyebrow incision (10 %), and body tattooing (16 %) were found to be widely practiced in Ethiopia.

**Conclusions:**

The overall pooled results of this study revealed that three out of ten children who underwent traditional uvulectomy experienced complications such as hemorrhage, the spread of contagious infections, and sepsis. Children underwent uvulectomy are also victim to other traditional malpractices including milk teeth extraction, bloodletting, eyebrow incision, tonsillectomy, and body tattooing. Efforts should be made to update strategies to avert malpractices through awareness-creation, social mobilization, and controlling traditional practitioners.

## 1 Introduction

Traditional uvulectomy is a potentially dangerous surgical procedure involves removing part or all of the uvula by traditional healers [1,2]. It is performed as part of local cultural beliefs usually among under-five children, and entails surgical resection of the uvula traditionally. The procedure includes pulling the tongue, with a string and then cutting it with a sharp object. This often uses unsterile materials and can lead to serious immediate and delayed complications. In Africa, neonatal uvulectomy by traditional practitioners has been an age-long practice [[3], [4], [5]]. Harmful traditional practice was deeply rooted in the community; more than 80 % of Middle East and European immigrants residents, still participated in this practice [6].

Different traditional malpractices are practiced (concurrently with uvulectomy) in Africa including Ethiopia. Female genital cutting is widely performed in Ethiopia and has a significant association with birth complications [[7], [8], [9], [10]]. Similarly, milk tooth extraction [11], eyebrow incision with uvula cutting [6] and bloodletting [12] were among some of the cultural malpractice in Ethiopia.

Even though it has varieties of complications traditional uvulectomy is still practiced in different African countries like Uganda [13], Tanzania [14], Kenya [15], Sudan [16], Cameroon [17], and others. The reasons for uvula removal in many African countries vary, but it is generally believed to cure various diseases by the community and traditional practitioners.

Uvulectomy is a dangerous harmful custom that have various complications among Ethiopian communities. The procedure lacks sterile technique and results in short and long-term complications like excessive bleeding, infections, the transmission of infectious disease, anemia, tetanus meningitis, and death [[18], [19], [20], [21], [22]]. Similarly, hospital admission secondary to sepsis and anemia after uvulectomy was a common problem in Ethiopia [23]. Additionally, acute malnutrition, human immune deficiency (HIV) infections, septicemia, dental complications, torticollis, difficulty swallowing, and obstructive sleep apnoea (secondary to palatal stenosis) were among commonly reported complications [[24], [25], [26], [27]].

Ethiopia has implemented a national strategy to put an end to all harmful malpractices, like uvulectomy, by 2025 [28]. This practice has been associated with various complications, and therefore, the country has made significant efforts to raise awareness about its negative consequences. Health education campaigns have been launched, and community mobilization efforts are underway to increase awareness among social help providers such as “Shengo” and religion front-runners, and malpractitioners. These efforts will attract customers and enhance service provision capacity, which is essential in achieving the national plan [[28], [29], [30]].

Despite those efforts, traditional uvulectomy is still widely practiced in Ethiopia [31]. There are different study reports on complications of uvulectomy and co-occurrences of other traditional malpractices in Ethiopia. A national summary of evidence that indicates the burden of uvulectomy complications and concurrent occurrences is needed to provide the reader with up-to-date information and support for those who are interested in tackling these traditional practices. Consequently, the study assessed the pooled traditional uvulectomy complications and concurrent occurrences of malpractices within Ethiopia. It sheds light on health implications and enriches the understanding of cultural traditions, ethics in healthcare, and global health discourse.

## 2 Methods

### 2.1 Study protocol and registration

The following steps were taken to conduct this systematic review and meta-analysis (SRMA): initially, articles has been searched, then the titles and abstracts were assessed, and finally, full texts were evaluated to determine if they are eligible. The Preferred Reporting Items for Systematic Review and Meta-analyses (PRISMA) guideline were followed [32] [Supplementary File 1]. Additionally, the Meta-analysis of Observational Studies in Epidemiology (MOOSE) criteria were applied to present the findings [33]. Registered on PROSPERO (CRD42023459734).

### 2.2 Inclusion of the studies

Ethiopian studies that reported the complications of traditional uvulectomy and/or co-occurrence of traditional malpractices were considered. This SRMA includes observational researchs, including cross-sectional, cohort, and case-control studies, that investigate the complications of uvulectomy and/or concurrent malpractices. The articles considered in this review were published in Ethiopia and available until June 1, 2023, and were written in English. The search for articles took place between August 20 and August 31, 2023. Note that this review does not include case series/reports, editorials, reviews, commentaries, or experimental investigations.

### 2.3 Study searching strategies

The systematic review and meta-analysis utilizes several databases including PubMed, EMBASE, CINHAL (EBSCO), Google Scholar, Web of Sciences, MEDLINE, Cochrane Library, SCOPUS, and Google search. Boolean logic operators (AND, OR, NOT), Medical Subject Headings (MeSH), and keywords were utilized to locate articles. Searching terms “Complications/risk” AND “Uvulectomy/uvula cutting” AND “co-occurrence/concurrent occurrences” AND “traditional practices/malpractices” AND “Ethiopia” were used to retrieve relevant articles.

Advanced PubMed searches utilize (((((((((((Uvulectomy) OR (“traditional uvulectomy")) OR (“Uvula cutting")) OR (“Uvula removal")) AND (Complications)) OR (Risks) AND (“Co-occurrence")) OR (“Concurrent occurrences")) AND (malpractices)) OR (“traditional malpractices")) OR (“Cultural malpractices")) AND (Ethiopia). The detail comprehensive searchs is located under “Supplementary File 2."

### 2.4 Study selection

**Fig. 1:**
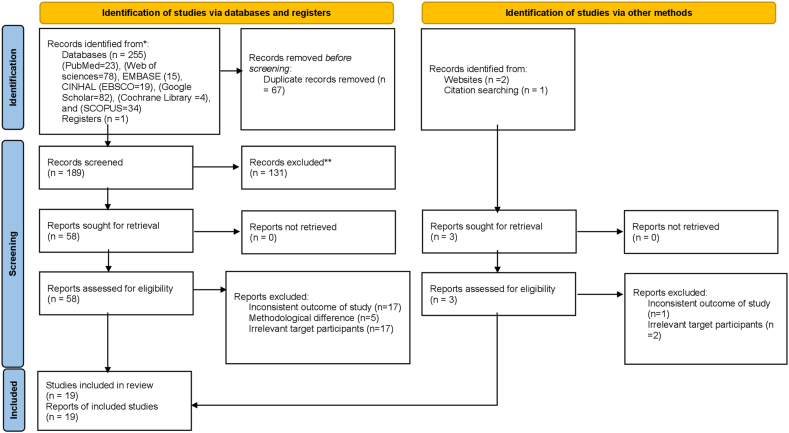
PRISMA statement flow diagram that display the study selection procedure.

EndNote X8 was mainly used to merge database search results and eliminate duplicates. However, some references were manually managed owing to variations in citation styles across various sources. Afterward, 3 authors (TG, AE, and AN) individually went through the titles and abstracts of the papers. The other two authors (SM and AC) examined the full texts and independently analyzed each study considering its objectives, research design, population, and primary outcomes. The main objective was to assess the complications of traditional uvulectomy and the occurrence of concurrent traditional malpractices in Ethiopia. The PRISMA flow diagram illustrates the entire study selection process (Fig. 1).

### 2.5 Abstraction of data

Following the identification of relevant articles, TG and AN extracted the data independently. They used a pre-defined Microsoft Excel 2016 sheet with headings: authors and study year, setting, area, design of study, sample size, the main outcome (complications of uvulectomy), and concurrent occurrences of other traditional malpractices to extract data from the selected papers. Quantitative data, including total sample size (N), frequency of occurrence (n), and effect size, were compiled using Microsoft Excel 2016. These data were taken from the included papers and used for pooled-analysis.

### 2.6 Study outcome

Complications arising from traditional uvulectomy procedures were the main results for the given SRMA, with secondary finding being the co-occurrence of traditional malpractices in Ethiopia.

### 2.7 Risk of bias

The Joanna Briggs Institute (JBI), the tool for assessment of quality was utilized and assessed the likelihood of bias in observational studies [34]. To minimize potential bias, comprehensive searches through manual and electronic/database searches, such as published/unpublished and institutional/community-based research were conducted. We authors collaborated to establish a timetable for selecting studies as per specific aims and suitability requirements, assessing quality of studies, routinely assessing review procedure, and gathering and extracting the data.

### 2.8 Methodological quality (risk bias) assessment

The JBI methodology was used to evaluate the quality of the research methods and findings in the selected observational studies, including cross-sectional, case-control, and cohort studies [34] ***[***Supplementary file 3***].*** Two independent groups of authors, TG and AN, and AE and SM, evaluated the quality of the studies. The final decision on study inclusion was based on the average score of the two groups. If there were disagreements about including a study, they were resolved through discussion. The studies were assessed against each criterion of the quality assessment tool and categorized as high, moderate, or low quality. A score above 80 % was considered high quality, while a score between 60 % and 80 % was considered moderate quality, and a score below 60 % was considered low quality. Only studies with a score of 60 % or higher were included in the analysis. The purpose of this quality assessment was to evaluate the internal validity (systematic error) and external validity (generalizability) of the studies and to minimize the risk of bias.

### 2.9 Statistical analysis and publication bias

STATA 17 software was used to synthesize data and conduct statistical analyses. The results of meta analysis, which were accustomed demonstrate the complications of uvulectomy and co-occurrences of traditional malpractices, were showed by forest plots. To reduce heterogeneity among the incorporated studies, a random effects model was utilized. Additionally, The data were analyzed by subgroups defined by different study variables including study settings, study environment (community-settings/facility-based), and years of publications. Finally, Meta-regression analysis was performed to explore heterogeneity sources.

A systematic synthesis of observational evidence was undertaken utilizing Higgins et al.'s I^2^ statistic. If the I^2^ value was equal to or higher than 75/100 %, it indicated significant heterogeneity. A funnel plot and Egger's regression test were used to assess publication bias. Asymmetry in the funnel plot and/or a statistically significant Egger's regression test (P < 0.05) indicated the presence of publication bias. The results of the included studies were initially summarized narratively, followed by a meta-analysis chart.

## 3 Results

### 3.1 Screening flow

After conducting various source searches, totally 259 studies were identified through extensive searchs, from different data sources. However, 67 of these were removed as duplicates. The rest 192 publications were filtered using study title and summaries. From these, 131 studies have been excluded, with 58 being rejected based on their titles, 69 after reviewing their abstracts, and 4 not conducted in Ethiopia. Other, 42 papers were also excluded (inconsistent outcome of study (n = 18), methodological difference (n = 5), irrelevant target participants (n = 19)). Ultimately, 19 publications fulfilled an inclusion standards and were retained for inclusion in the review (Fig. 1).

### 3.2 Characteristics of the studies

This meta-analysis and systematic review included 19 papers with a total of 23559 study participants [6,21,23,30,[35], [36], [37], [38], [39], [40], [41], [42], [43], [44], [45], [46], [47], [48], [49]], of which 11354 children underwent uvulectomy. Nearly fifty percent of the research studies included in this review were carried out in the Amhara [21,23,30,35,37,39,40,43,49], followed by Tigray (21 %) [6,38,41,42], Oromia (10 %) [36,45], and Southern Nations, Nationalities and Peoples Regional state (SNNPR) regions (10 %) [44,46]. The remaining 10 % of studies were national [47,48]. Except for one study which had a prospective cohort design, all others were cross-sectional [46]. In review, half of the investigations were carried out in hospital settings, and the other half were conducted in community settings. One study was carried out in a community and an institutional context. A summary of the key characteristics of the papers that were chosen for this review is found in Table 1.

**Table 1:** General characteristics of included studies that reported complications of traditional uvulectomy and co-occurrences of traditional malpractices in Ethiopia.

Author, Year	Region	Setting	Study design	sample size	Prevalence	Complications of traditional Uvulectomy	Co-occurrences of other traditional malpractices
Abera B, 2014	Amhara	Hospital based	cross-sectional	253	21	Transmission of communicable infections (HIV, Hep B)	Female genital cutting (FGC): (20.8 %) of the children who had had uvulectomy were also victim for FGC)
Addis G, 2002	Oromia	Institution and community based	cross-sectional	80	37.5	NR	Concurrent to traditional uvulectomy, other malpractices like FGC (55 %), Milk tooth extraction(32 %), bloodletting/mebtat (7.5 %) were also practiced
Bayih A, 2020	Amhara	Institution-based	cross sectional	422	15.9	Transmission of communicable infections (HIV, Hep B), Hemorrhage, wound infection, fever, difficulty of breathing, and Tetanus	NR
Dagnew M, 1990	Amhara	Institution-based	cross-sectional	853	85.7	wound infection, fever, difficulty of breathing, and Tetanus	Milk -teeth extraction (70.2 %), Eyelid incision (19.2 %), Female circumcision (6.1 %)
Gebrekirstos K, 2014	Tigray	Community based	cross sectional	752	86.9	Wound infection, fever, difficulty of breathing, and Tetanus	Milk teeth extraction (12.5 %), eye borrows incision (2.4 %), FGM (0.7 %), bloodletting (2.13 %)
Gebrekirstos K, 2014	Tigray	Community based	cross sectional	752	72.9	Hemorrhage, wound infection, fever, difficulty of breathing, and Tetanus	Milk teeth extraction (11.8 %), eye borrows incision (2.3 %), FGM (0.7 %), bloodletting (2.13 %)
Gedefaw G, 2019	Amhara	Institution-based	cross-sectional	338	28.7	Transmission of communicable infections (HIV, Hep B)	NR
Kiflu G, 2014	Amhara	Community based	cross-sectional	503	22.9	NR	Milk teeth extraction (10.9 %), FGC 3 (1.3 %)
Getu D, 2010	Amhara	Community based	cross-sectional	1747	89	NR	Milk-teeth extraction (58.2), Eyebrow incision (16.7 %), bloodletting (2.3 %), FGC (4.3 %), Tonsillectomy (20 %)
Hadush A, 2016	Tigray	Institution-based	cross-sectional	290	20	NR	NR
Hailu A, 2019	Tigray	Community based	cross-sectional	1298	71.6	NR	NR
Kebede K, 2017	Amhara	Community based	cross-sectional	456	23.7	Hemorrhage, wound infection, fever, difficulty of breathing, and Tetanus	NR
Kefelew E, 2023	SNNPR	Institution-based	cross-sectional	402	36.6	wound infection, fever, difficulty of breathing, and Tetanus	NR
Mamuye B, 2020	Oromia	Institution-based	cross-sectional	363	12.4	Transmission of communicable infections (HIV, Hep B)	FGC (77.1 %), Body tattooing (5.7 %)
Medhin G, 2010	SNNPR	Community based	prospective Cohort	1799	4.8	NR	FGC (1.5 %), milk teeth extraction (3.9 %)
Mitke Y, 2010	National	Institution-based	cross-sectional	1163	19.9	Transmission of communicable infections (HIV, Hep B)	Milk tooth extractions (16 %), tonsillectomy (11.6 %)
Sentjens R, 2002	National	Institution based	cross-sectional	4830	42.2	Transmission of communicable infections (HIV, Hep B)	Eye brow incision (11.5 %), FGC (91.7 %), Tattooing (22.1 %)
Tadesse T, 2011	Amhara	Community based	cross-sectional	6624	85.5	NR	Milk teeth extraction (42.3 %), tattooing (21.5 %), and FGC (1.1 %)
Yirdaw B, 2022	Amhara	Community based	cross-sectional	634	52.5	wound infection, fever, difficulty of breathing, and Tetanus	NR

∗ AOR = adjusted odds ratio, NR: not reported, SNNPR = southern nations, nationalities and peoples region.

### 3.3 Complications of traditional uvulectomy

**Fig. 2:**
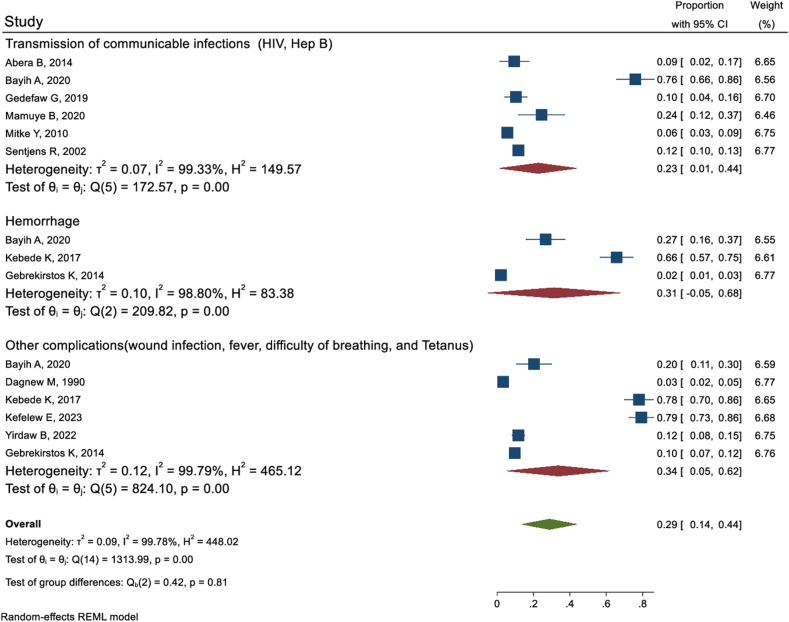
Systematic and Meta-analysis review showing complications of traditional uvulectomy in Ethiopia.

A meta-analysis found the overall prevalence of uvulectomy/uvula cutting in Ethiopia found to be 44 %. For a comprehensive understanding of the pooled prevalence and associated factors, please refer to Ref. [31]. Of nineteen studies reported about traditional practices in Ethiopia ten of them reported the complications of traditional uvulectomy. Based on those ten studies [21,23,30,35,37,[43], [44], [45],^47^,^48^], the pooled incidence of complications following traditional uvulectomy in Ethiopia was 29 % (95 % CI: 14%–44 %). Hemorrhage as a complication of traditional uvulectomy was reported by three studies [23,38,43]. The pooled incidence of hemorrhage was 31 % (95 % CI: 5%–68 %). Six studies [21,23,35,45,47,48] reported that traditional uvulectomy risked victims to communicable infections like HIV and Hepatitis virus mainly through contaminated devices used for this procedure. The Pooled result revealed that 23 % (95 % CI: 1%–44 %) of victims encountered communicable infections including HIV and Hepatitis virus. The pooled result from other six studies [6,23,30,37,43,44] also revealed that 34 % (95 % CI: 5%–62 %) of children exposed to traditional uvulectomy developed one or more complications like wound infection, fever, difficulty of breathing, and tetanus (Fig. 2).

### 3.4 Concurrent traditional malpractices

#### 3.4.1 Female genital cutting (FGC)

**Fig. 3:**
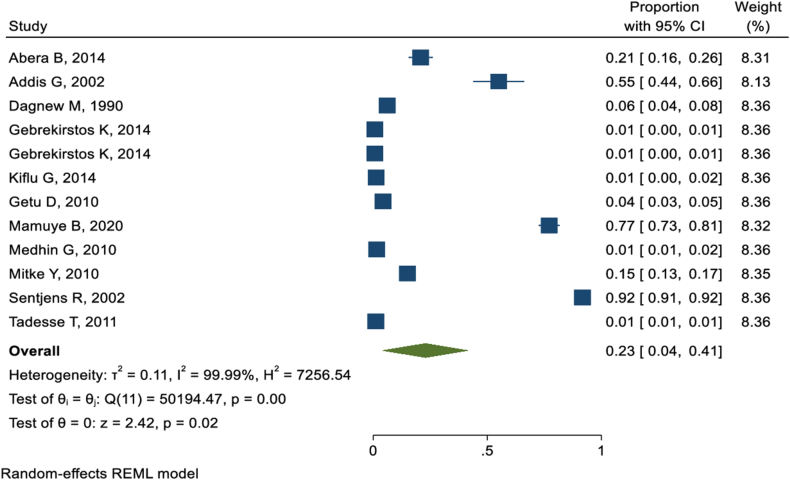
Meta-analysis of FGC prevalence in individuals with a history of traditional uvulectomy, visualized in a forest plot.

Traditional malpractices are mostly practiced simultaneously. A total of twelve studies [6,[35], [36], [37], [38], [39], [40],[45], [46], [47], [48], [49]] reported that FGC was practiced concurrently with traditional uvulectomy. The pooled prevalence of FGC among participants who were victims of traditional uvulectomy was 23 % (95 % CI: 4%–41 %) (Fig. 3).

#### 3.4.2 Milk tooth extraction

**Fig. 4:**
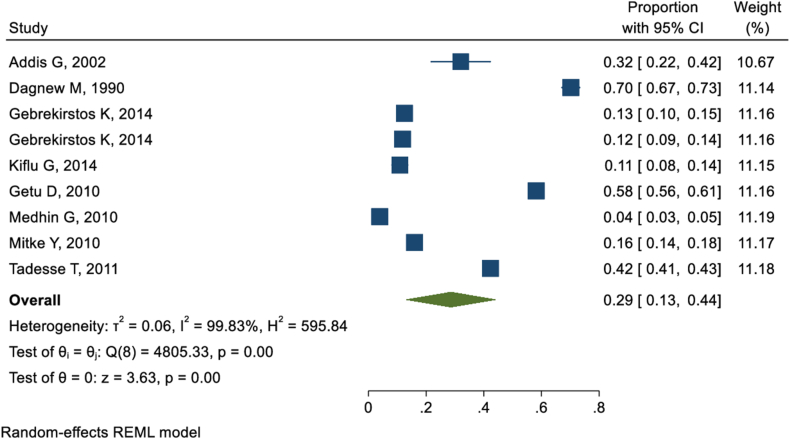
Forest-plot presentation of magnitude of milk tooth extraction concurrent to traditional uvulectomy.

Nine studies [[36], [37], [38], [39], [40],^46^,^47^,^49^,^50^] revealed that milk tooth extraction was practiced concurrently with traditional uvulectomy in Ethiopia. The pooled concurrent practice of milk tooth extraction in Ethiopia was found to be 29 % (95 % CI: 13%–44 %) (Fig. 4).

#### 3.4.3 Bleeding/bloodletting (mebtat in Amharic)

**Fig. 5:**
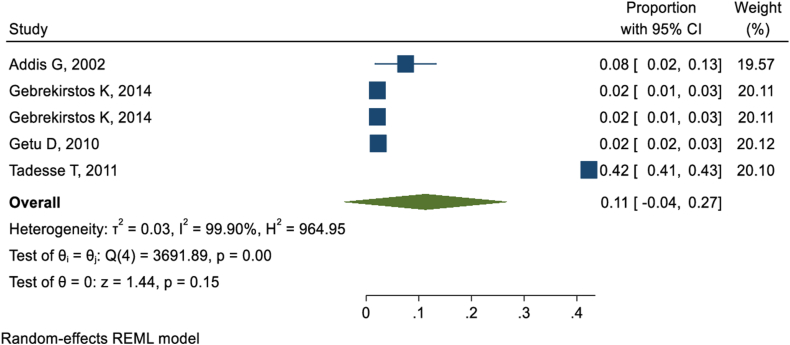
Forest-plot indicating magnitude of bloodletting practice concurrent to traditional uvulectomy.

Bloodletting, what we call “mebtata” in Amharic, was also practiced simultaneously among victims of traditional uvulectomy as reported by five studies [6,36,38,40,49] conducted on traditional uvulectomy. Thus, the pooled prevalence of bloodletting/bleeding practice among those who encountered traditional uvulectomy/uvula cutting was 11 % (95 % CI: 11%–27 %) (Fig. 5).

#### 3.4.4 Eyebrow incision, tattooing, and tonsillectomy

**Fig. 6:**
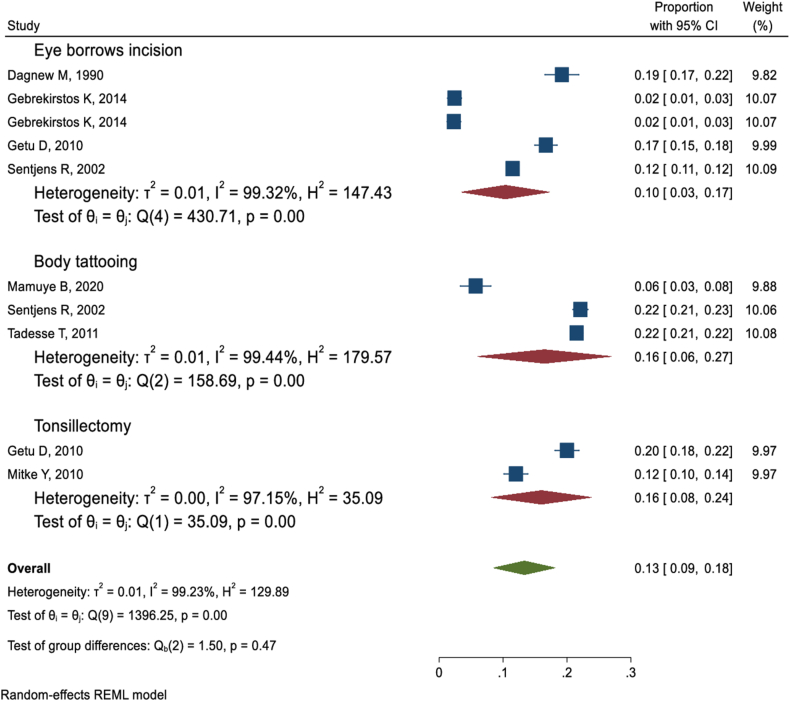
Forest plot of factors associated with uvulectomy.

Simultaneous to traditional uvulectomy other traditional malpractices including eyebrow incision, body tattooing, and tonsillectomy were reported by five [6,37,38,40,48], three [45,48,49], and two [40,47] studies, respectively. The pooled prevalence of eyebrow incision, body tattooing, and tonsillectomy among those who were victims of traditional uvulectomy was 10 % (95 % CI: 3%–17 %), 16 % (95 % CI: 6%–27 %), and 16 % (95 % CI: 8%–24 %), respectively (Fig. 6).

### 3.5 Factors driving uvulectomy practice in Ethiopia

Seven studies [6,30,38,39,43,44,47] conveyed the primary reasons why mothers opt for a traditional uvulectomy. These studies involved 4662 mothers, out of which 2136 mothers gave one or more reasons related to uvulectomy. The commonly cited reasons include preventing uvula swelling, pus, and rapture [6,38,39,43], improving medical treatment [30,38], avoiding sore throats [30,47], avoiding coughs [43,47], due to religious beliefs [43], and pressure from traditional practitioners [44,47]. It was commonly believed that a uvulectomy was necessary for infants who could not eat, exhibited an abnormal presentation of the anterior fontanel, spoke harshly, salivated excessively, and feared tonsil/uvula rupture [47]. Additional reasons mentioned by participants were prior success, fever, coughing, sneezing, and fatigue [43,44,47].

### 3.6 Heterogeneity and publications biases

**Fig. 7:**
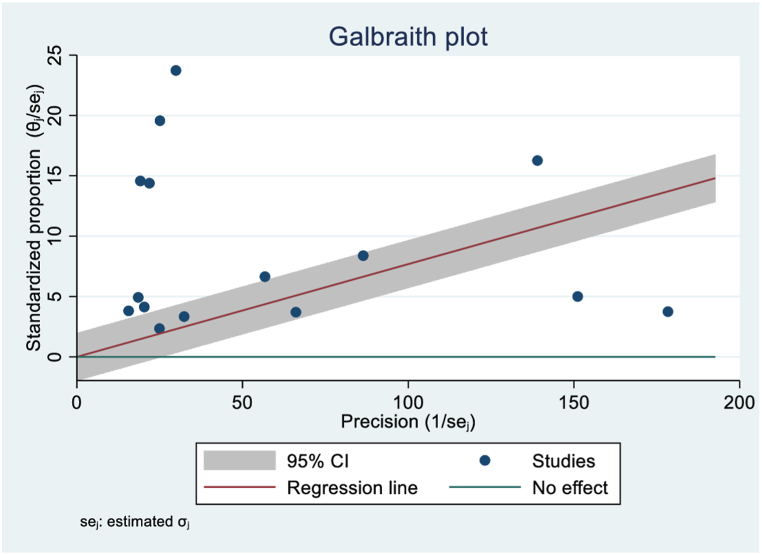
Visualization of study heterogeneity.

**Fig. 8:**
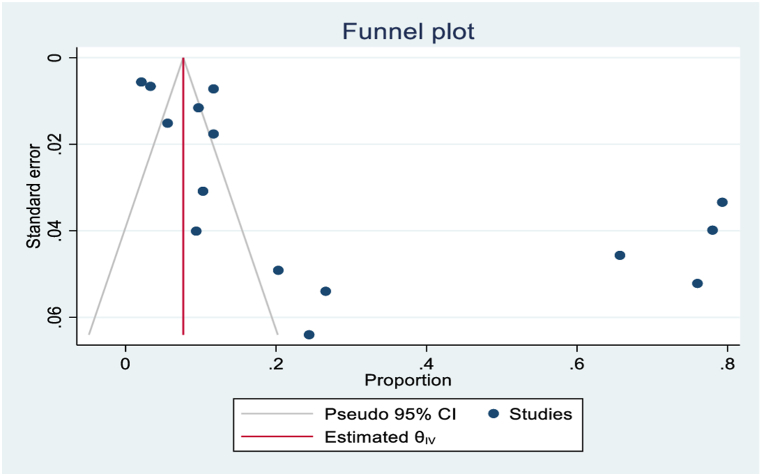
Funnel diagram/plot reviewed articles.

To evaluate potential publication bias in the included studies, funnel plots and Egger's tests were conducted at a 5 % significance level, accounting for heterogeneity. Moreover, Galbraith plots were used to identify heterogeneity sources based on factors including sample size and publication year (Fig. 7). The presence of publication bias was confirmed by Egger's test suggesting that studies with non-significant or negative results may have been underreported, as shown by ‘Fig. 8’ asymmetric funnel plot. Random-effects sensitivity analyses were conducted to assess the influence of individual studies on the overall pooled outcome. However, these analyses did not reveal substantial evidence of such an impact.

## 4 Discussion

Traditional/cultural uvulectomy/uvula cutting is a longstanding tradition in Africa, with different views regarding the reasons and its benefits [23,51]. However, one of the national plans to avert deaths of newborns and under 5 children is eradicating traditional malpractices and their complications by 2025 [52]. The 2025 national strategy aims to address harmful traditional practices (HTPs), through awareness raising, establishing and updating of practitioners' registry, informing them about relevant laws, and providing training and encouraging these practitioners to become change agents, engaging community leaders, and taking legal measures against those who continue to practice secretly. This comprehensive approach aims to reduce HTPs and promote safer, lawful practices within the community.

Traditional uvulectomy is a common and hazardous procedure that poses a significant life-threatening consequence to children in Ethiopia. This study investigated the incidence of different complications following traditional uvulectomy and the co-occurrence of uvulectomy with many other traditional malpractices. Consequently, the pooled incidence of uvulectomy complications in Ethiopia was found to be 29 % (95 % CI: 14%–44 %). The summary estimate of this study revealed that hemorrhage (31 %), transmission of communicable infections including HIV and Hepatitis virus (23 %), and sepsis (34 %) are the most common complications of traditional uvulectomy mainly due to the use of unsterile instruments and exposure to blood. Traditional practitioners often lack infection control knowledge, and protective measures are rarely used. Cultural beliefs and limited healthcare access perpetuate these practices. Education, safer practices, and training in hygiene are essential to reduce these risks.

This review, pooled estimate of hemorrhage following uvulectomy/uvula cutting found to be 31 % (95 % CI: 5%–68 %). Similarly, hemorrhage as a complication of uvulectomy has also been reported in Nigeria [53] and Tanzania [54]. This finding implied that despite the efforts of non-governmental and governmental organizations in increasing access to health care, dissemination of information about harmful traditional practices, and setting legal actions for those who perform, traditional uvulectomies remain widespread and prevalent. This indicated that there is a need to design an innovative approach that can lessen the impact of uvulectomy and its consequences. Furthermore, to save the lives of those children, there should be a system which aware of those complications and can give prompt treatment, especially in the area where traditional uvulectomy is prevalent.

In this SRMA study, the overall estimate of sepsis following uvulectomy was 34 % (95 % CI: 5%–62 %). Study in Nigeria also have documented cases of sepsis following traditional uvulectomy [55]. Sepsis occurred mainly due to the materials used for cutting of uvula were sterile. Research done in Uganda indicated that sharp blade wetlands, strings, electrical lines, reaper blades, and utensils are among the instruments used to cut uvula [56]. This result still indicated that traditional uvulectomy poses significant consequences for the children. Therefore, it is necessary to amend strategies to combat the traditional harmful practices even if it is necessary to formulate a strong rule and regulations for those practitioners and families.

This study revealed that the pooled co-occurrence of female genital cutting (FGC) and traditional uvulectomy was 23 % (95 % CI: 4%–41 %). A study conducted in Nigeria similarly reported that children suffered from both uvulectomy and FGC [57]. Female genital circumcision is a common traditional malpractice in Ethiopia which is usually performed with other traditional malpractices like uvulectomy. Evidence shows that the rate of FGC in Ethiopia is from 14 to 24 % [52]. Women who cut their female genitalia suffer physical health consequences for the rest of their lives, starting when they cut as babies and continuing through adult sexuality and motherhood. Therefore, the concerned bodies should strive hard to keep those children from the unwanted consequences of those traditional malpractices.

Similarly, the pooled estimate discovered traditional malpractices including milk tooth extraction (29 %), bloodletting (“mebtata” in Amharic) (11 %), eyebrow incision (10 %), and body tattooing (16 %) were widely practiced concurrently with traditional uvulectomy in Ethiopia. This indicates that if the current situation continues, Ethiopia will not be able to achieve its national goal of eliminating all traditional harmful practices by 2025 [52].

### 4.1 Implication

This review is pioneering to provide a thorough evaluation of the risks and co-occurrences of typical malpractices associated with uvulectomy. The data gathered from this review is helpful for healthcare practitioners, government decision-makers, and other organizations committed to improving children's health by providing a national summary estimate of the prevalences of complications following cultural uvulectomy/uvula cutting and co-occurrences of malpractices. It can help translate health-related evidence, support informed decision-making, and compile a summary of data into a single document.

### 4.2 Strengths and limitations

Among the notable strengths of this review are use of multiple databases for manual and electronic article searches to prepare for meta-analyses. It also used a predetermined and validated standard format for uniform information abstraction. This format was reviewed by two separate reviewers to reduce errors. Moreover, papers from different regions of the country with both urban and rural populations were included in this meta-analysis. As a limitation: When applying the review's findings, it is important to keep in mind that the high degree of heterogeneity among the included studies may have resulted in inadequate power to identify statistically significant relationships.

## 5 Conclusions

Complications of traditional uvulectomy and concurrent malpractices pose significant consequences for the children. The pooled results of this SRMA revealed that three out of ten children who underwent this procedure experienced complications such as hemorrhage, the spread of contagious infections, and sepsis. In addition to uvulectomy, concurrently performed traditional practices on Ethiopian children include extraction of milk teeth, bloodletting (known as “mebtat” in Amharic), eyebrow incision, tonsillectomy, and body tattooing. Therefore, it is necessary to establish strict policies and requirements for traditional practitioners and families too. Additionally, implementing education campaigns to inform communities about the risks of traditional malpractices and the benefits of modern medicine. Establish and update a registry of practitioners, enforce laws against harmful practices, and provide alternative employment training. Engaging community leaders as change agents and developing support systems like health clinics are recommended.

## Ethical considerations

In this study, no ethical approval was needed as the study was based on data extracted from published studies.

## Funding statements

None.

## Data availability statements

The data associated with this review can be found in the results section/supplementary materials/referenced articles.

## CRediT authorship contribution statement

**Tamirat Getachew:** Writing – review & editing, Writing – original draft, Visualization, Validation, Supervision, Software, Resources, Project administration, Methodology, Investigation, Formal analysis, Data curation, Conceptualization. **Abraham Negash:** Writing – review & editing, Writing – original draft, Supervision, Resources, Methodology, Investigation, Data curation, Conceptualization. **Sinetibeb Mesfin Kebede:** Writing – review & editing, Writing – original draft, Visualization, Supervision, Software, Project administration, Methodology, Data curation. **Abera Cheru:** Writing – review & editing, Writing – original draft, Visualization, Validation, Supervision, Resources, Methodology, Investigation. **Addis Eyeberu:** Writing – review & editing, Writing – original draft, Validation, Software, Resources, Project administration, Methodology, Investigation, Data curation. **Abera Kenay Tura:** Writing – review & editing, Writing – original draft, Validation, Supervision, Resources, Formal analysis, Data curation.

## Declaration of competing interest

The authors declare that they have no any competing interests.

## Footnotes

Appendix A Supplementary data to this article can be found online at https://doi.org/10.1016/j.heliyon.2024.e38978.
