# Supplementary material for: The complications of traditional uvulectomy and concurrent occurrences of cultural malpractices in Ethiopia: A systematic review and meta-analysis
Source: Heliyon. 2024 Oct 5;10(19):e38978. doi: 10.1016/j.heliyon.2024.e38978 (PMC11492597; doi:10.1016/j.heliyon.2024.e38978)
Supplement: Multimedia component 1 [file mmc1.docx]

Searching strategy for the complications of traditional uvulectomy and concurrent occurrence of traditional malpractices in Ethiopia: a systematic review and meta-analysis

| Databases | Searching terms | Number of studies |
| --- | --- | --- |
| PubMed | (((((((((((Uvulectomy) OR ("traditional uvulectomy")) OR ("Uvula cutting")) OR ("Uvula removal")) AND (Complications)) OR (Risks) AND ("Co-occurrence")) OR ("Concurrent occurrences")) AND (malpractices)) OR ("traditional malpractices")) OR ("Cultural malpractices")) AND (Ethiopia) | 23 |
| Google scholar | Complications/risk and Uvulectomy/uvula cutting and co-occurrence/ concurrent occurrences and traditional practices/ malpractices and Ethiopia | 82 |
| SCOPUS | (uvulectomy) OR (uvula cutting) OR (uvulectomy AND complications) OR (uvula cutting AND concurrent occurrences) OR (uvulectomy AND co-occurrences) AND (Ethiopia) | 34 |
| Web of Science | (uvulectomy) OR (uvula cutting) OR (uvulectomy AND complications) OR (uvula cutting AND concurrent occurrences) OR (uvulectomy AND co-occurrences) AND (Ethiopia) | 78 |
| Others databases |  | 42 |
| Total retrieved |  | 259 |
| Included |  | 19 |
