# Supplementary material for: The complications of traditional uvulectomy and concurrent occurrences of cultural malpractices in Ethiopia: A systematic review and meta-analysis
Source: Heliyon. 2024 Oct 5;10(19):e38978. doi: 10.1016/j.heliyon.2024.e38978 (PMC11492597; doi:10.1016/j.heliyon.2024.e38978)
Supplement: Multimedia component 2 [file mmc2.docx]

**Appraisal**

Critical appraisal check list of quantitative studies on complications of traditional uvulectomy and co-occurrences of traditional malpractices in Ethiopia.

(1 =yes, 0=no/not mentioned); total score=8

| **Studies** | **Q1** | **Q2** | **Q3** | **Q4** | **Q5** | **Q6** | **Q7** | **Q8** | **Total score** | **Remark** |
| --- | --- | --- | --- | --- | --- | --- | --- | --- | --- | --- |
| Abera B, 2014 | Y | Y | Y | N | N | N | Y | Y | 5/8 | Has different outcome of interest |
| Addis G, 2002 | Y | Y | Y | Y | Y | N | Y | Y | 7/8 |  |
| Bayih A, 2020 | Y | Y | Y | Y | Y | Y | Y | Y | 8/8 |  |
| Dagnew M, 1990 | Y | Y | Y | Y | U | N | Y | Y | 6/8 | Has different outcome of interest |
| Gebrekirstos K, 2014 | Y | Y | Y | Y | Y | Y | Y | Y | 8/8 |  |
| Gebrekirstos K, 2014 | Y | Y | Y | Y | Y | Y | Y | Y | 8/8 |  |
| Gedefaw G, 2019 | Y | Y | Y | Y | Y | Y | Y | Y | 8/8 |  |
| Kiflu G, 2014 | Y | Y | Y | Y | Y | Y | Y | Y | 8/8 |  |
| Getu D, 2010 | Y | Y | Y | Y | Y | Y | Y | Y | 8/8 |  |
| Hadush A, 2016 | Y | N | Y | U | Y | Y | Y | Y | 6/8 |  |
| Hailu A, 2019 | Y | Y | Y | N | Y | N | Y | Y | 6/8 |  |
| Kebede K, 2017 | Y | Y | Y | Y | Y | Y | Y | Y | 8/8 |  |
| Kefelew E, 2023 | Y | Y | Y | Y | Y | Y | Y | Y | 8/8 |  |
| Mamuye B, 2020 | Y | Y | Y | Y | Y | N | U | Y | 6/8 | Has different outcome of interest |
| Medhin G, 2010 | Y | Y | Y | Y | N | Y | Y | Y | 7/8 |  |
| Mitke Y, 2010 | Y | Y | Y | Y | Y | Y | Y | Y | 8/8 |  |
| Sentjens R, 2002 | Y | N | Y | Y | Y | Y | Y | Y | 7/8 |  |
| Tadesse T, 2011 | Y | Y | Y | Y | Y | N | Y | Y | 7/8 |  |
| Yirdaw B, 2022 | Y | Y | Y | Y | Y | Y | Y | Y | 8/8 |  |

Notes:

Q1 - Were the criteria for inclusion in the sample clearly defined?

Q2 - Were the study subjects and the setting described in detail?

Q3 - Was the exposure measured in a valid and reliable way?

Q4 - Were objective, standard criteria used for measurement of the condition?

Q5 - Were confounding factors identified?

Q6 - Were strategies to deal with confounding factors stated?

Q7 - Were the outcomes measured in a valid and reliable way?

Q8 - Was appropriate statistical analysis used?

**Abbreviations**: Y, yes; N, no; U, unclear.
